# BGWAS: Bayesian variable selection in linear mixed models with nonlocal priors for genome-wide association studies

**DOI:** 10.1186/s12859-023-05316-x

**Published:** 2023-05-11

**Authors:** Jacob Williams, Shuangshuang Xu, Marco A. R. Ferreira

**Affiliations:** Department of Statistics, Virginia Tech, Blacksburg, 24061 USA

**Keywords:** GWAS, Bayesian, Model selection

## Abstract

**Background:**

Genome-wide association studies (GWAS) seek to identify single nucleotide polymorphisms (SNPs) that cause observed phenotypes. However, with highly correlated SNPs, correlated observations, and the number of SNPs being two orders of magnitude larger than the number of observations, GWAS procedures often suffer from high false positive rates.

**Results:**

We propose BGWAS, a novel Bayesian variable selection method based on nonlocal priors for linear mixed models specifically tailored for genome-wide association studies. Our proposed method BGWAS uses a novel nonlocal prior for linear mixed models (LMMs). BGWAS has two steps: screening and model selection. The screening step scans through all the SNPs fitting one LMM for each SNP and then uses Bayesian false discovery control to select a set of candidate SNPs. After that, a model selection step searches through the space of LMMs that may have any number of SNPs from the candidate set. A simulation study shows that, when compared to popular GWAS procedures, BGWAS greatly reduces false positives while maintaining the same ability to detect true positive SNPs. We show the utility and flexibility of BGWAS with two case studies: a case study on salt stress in plants, and a case study on alcohol use disorder.

**Conclusions:**

BGWAS maintains and in some cases increases the recall of true SNPs while drastically lowering the number of false positives compared to popular SMA procedures.

**Supplementary Information:**

The online version contains supplementary material available at 10.1186/s12859-023-05316-x.

## Background

Genome-wide association studies (GWAS) are a popular tool to identify causal relationships between variations in the genome and observed phenotypes. In GWAS studies, the most commonly considered genomic variations are single nucleotide polymorphisms (SNPs), which may be of the order of 100,000–1,000,000 s depending on the species and the dataset. An important aspect of GWAS analysis is the existence of correlation among the observations as a result of study design or population structure. A popular way to deal with this correlation is to use linear mixed models that include kinship random effects with a covariance matrix proportional to a realized relationship matrix [1–3]. The most widely used procedures for GWAS analysis are single marker association tests (SMA), which evaluate the individual predictive ability of each SNP by fitting as many linear mixed models (LMMs) as the number of SNPs [1], each model only containing one SNP. In a traditional SMA, after evaluating each SNP individually, a multiple comparison correction, such as the Bonferroni correction or the Benjamini Hochberg correction, is used to identify important SNPs and attempt to control the false discovery rate (FDR). However, these SMAs based on LMMs still yield high FDR because the SNPs themselves are highly correlated [4]. To have better FDR control and still maintain the same ability to detect true positive SNPs, we propose a novel Bayesian method for linear mixed models with nonlocal priors for efficient analysis of GWAS data.

We call our novel method BGWAS. BGWAS has two steps: screening and model selection. First, the screening step fits as many LMMs as the number of SNPs, uses a mixture of a Dirac delta at zero and a nonlocal prior, and estimates the probability of the Dirac delta component. After that, the screening step computes the posterior probability of each SNP being a null SNP and uses Bayesian false discovery control [5–8] to choose a set of candidate SNPs. Second, the model selection step performs a model search where the possible models contain any number of SNPs from the set of candidate SNPs. When the model space is too large for complete enumeration, the BGWAS model selection step searches through the model space with a genetic algorithm (GA). A simulation study presented in the “Results” section shows that, when compared to SMA, BGWAS reduces the number of false positives while maintaining the same level of true casual SNPs recall.

**Fig. 1:**
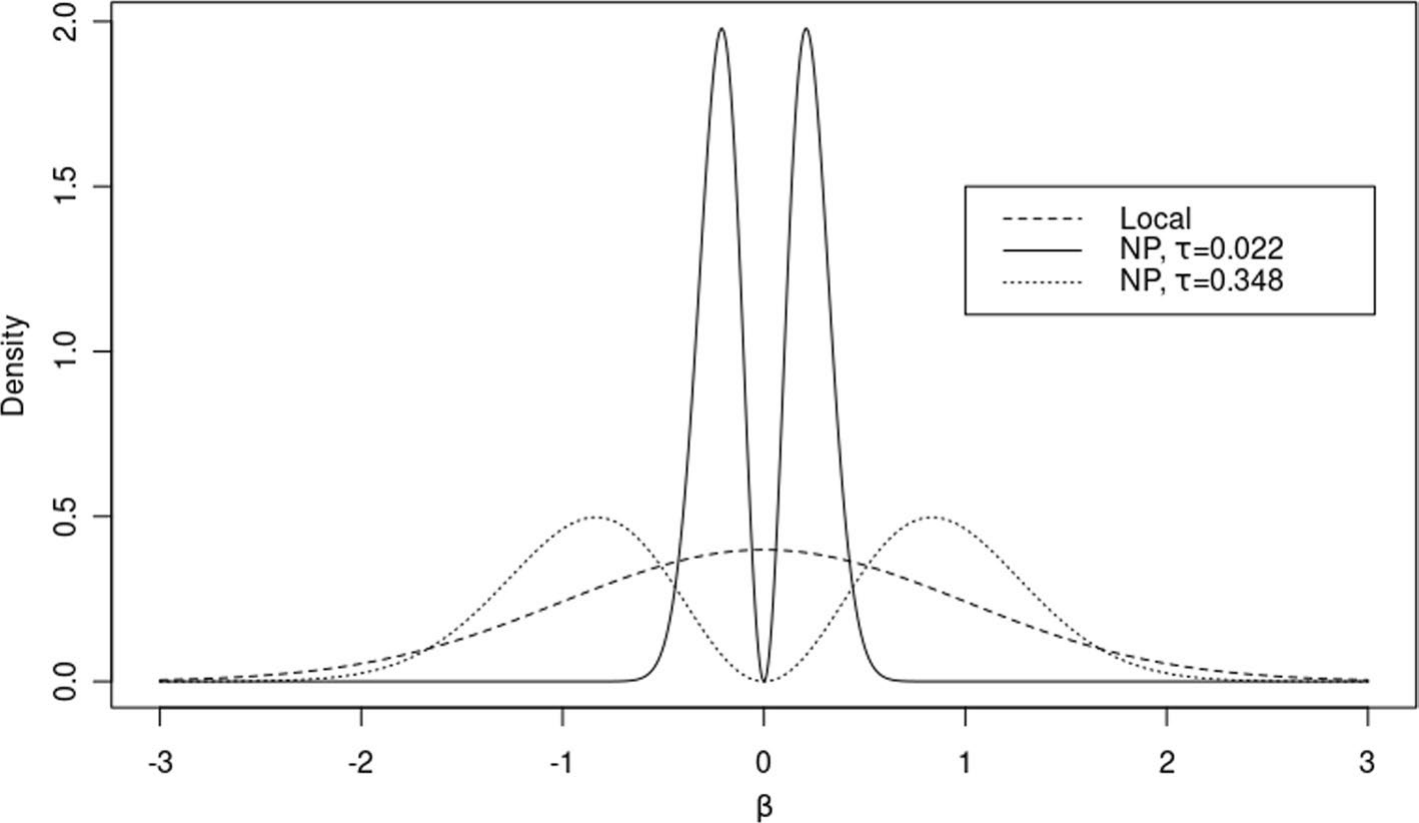
pMOM nonlocal priors with $\tau = 0.022$ and $\tau = 0.348$, as well as a local prior

BGWAS uses novel nonlocal priors specifically tailored for LMMs. Nonlocal priors were first proposed by [9] and extended fully to Gaussian linear models in [10]. [10] proposed product moment (pMOM) priors that are proportional to a Gaussian kernel multiplied by the product of the absolute values of the coefficients raised to a scalar. Figure 1 presents two pMOM priors and a local prior. When compared to local priors, nonlocal priors lead to a much faster accumulation of evidence in favor of a true null hypothesis [9, 10]. This property is especially useful in GWAS where the vast majority of SNPs are usually not important. [11] extended the pMOM nonlocal prior to generalized linear models by using a Gaussian kernel with a covariance matrix proportional to the diagonal of the Fisher information. In contrast, here we propose a pMOM nonlocal prior for LMMs that uses the full Fisher information matrix. When compared to using just the diagonal of the Fisher information matrix, the use of the full Fisher information matrix in the definition of the nonlocal prior better accounts for the correlations between SNPs and, thus, better controls the FDR.

Many of the published works regarding Bayesian analysis of GWAS data use Markov chain Monte Carlo (MCMC). [12] proposes a screening algorithm that identifies causal SNPs using local priors, but does not take into account the relationships between SNPs. Similarly, [13] uses local priors with a MCMC implementation in a screening algorithm to identify SNPs, but, similarly does not take into account the relationships between SNPs. [14] and [15] both propose two-step procedures, first screening the SNPs to reduce the size of the problem, and second using a model selection step with different local priors in MCMC implementations to identify causal SNPs. [14] does not take into account the kinship correlation structure among observations. [16] takes into account the correlation among observations and SNPs but uses local priors in both steps of an iterative two-step procedure. [17] proposes an iterative two-step procedure using $R^2$ and nonlocal priors in an MCMC implementation but does not take into account the kinship correlation structure. By not taking into account the kinship correlation structure, an increase of false positives is typically seen [1–3]. In contrast, our method BGWAS performs a Bayesian procedure using nonlocal priors that takes into account the kinship correlation structure and the relationships between SNPs. Importantly, instead of MCMC, BGWAS uses a fast Empirical Bayes procedure that analyzes GWAS problems of size $10^5$ to $10^6$ SNPs in a reasonable amount of time.

To decrease the computational burden of LMMs, BGWAS uses estimates of the variance components from baseline models for both the screening step and model selection step. Methods such as EMMAX [3] and population parameters previously determined (P3D [18]) have popularized estimating variance components from a baseline model in a SMA using LMMs. EMMAX avoids the repeated estimation of the variance components by using the heritability estimate from the null model for all SNPs. P3D uses both the estimate of the heritability and the estimate of the independent error structure parameter fixed while testing all SNPs. Similarly to EMMAX, BGWAS estimates the kinship dependence parameter from a baseline model. As EMMAX and P3D have shown, using variance estimates from a baseline model provides orders of magnitude faster results while losing little to no statistical power.

The remainder of this article is organized as follows. The Methods section presents our proposed BGWAS method for fast Bayesian SNP search. The Results section presents simulation results using genotype data from Illumina sequencing of 2772 humans as well as two case studies based on real world examples. The Conclusion and Future Directions section discusses conclusions and possible avenues of future research.

## Methods

BGWAS works in two distinct steps: a screening step and a model selection step. First, the screening step fits as many LMMs as the number of SNPs using a mixture of a Dirac delta at zero and a nonlocal prior, and estimates the probability of the Dirac delta component. After that, the screening step computes the posterior probability of each SNP being a null SNP and uses Bayesian false discovery control [5–8] to choose a set of candidate SNPs. Next, the BGWAS model selection step takes the set of candidate SNPs identified in the screening step and uses a novel multivariate nonlocal prior to perform Bayesian model selection among them. The goal of the model selection step is to further control false positives.

The model used in both the screening step and the model selection step is [1]

1 $$\begin{aligned} {{\textbf {Y}}} = X_c \varvec{\alpha }+ X_s\varvec{\beta }+ Z{{\textbf {u}}} + \varvec{\epsilon }\quad \text {where} \quad \varvec{\epsilon }\sim N(0, \sigma ^2 I) \quad \text {and} \quad {{\textbf {u}}} \sim N(0,\sigma ^2 \kappa K), \end{aligned}$$

where ${{\textbf {Y}}}$ is an $n \times 1$ phenotype vector, $X_c$ is an $n \times l$ matrix with columns including the intercept and fixed effects, $\varvec{\alpha }$ is an $l \times 1$ vector of regression coefficients, $X_s$ is an $n \times p$ matrix with columns including SNPs, $\varvec{\beta }$ is a $p \times 1$ vector of regression coefficients, *Z* is an $n \times t$ incidence matrix mapping each observed phenotype to one of *t* inbred strains, ${{\textbf {u}}}$ is a $t \times 1$ vector of random effects accounting for population structure, and $\varvec{\epsilon }$ is an error term. *K* is the realized relationship matrix or kinship matrix assumed to be a known positive semi-definite matrix calculated at the beginning of the procedure.

The remainder of this section is divided into two subsections: BGWAS Screening Step provides details about the screening step and BGWAS Model Selection Step presents the model selection step.

### BGWAS screening step

The screening step fits as many LMMs as the number of SNPs, with each LMM having only one SNP in addition to the control regressors. To speed up computations, we use an approach similar to P3D, which is widely used in SMA for GWAS [3, 18]. Specifically, the variance parameter $\kappa$ and the vector of coefficients $\varvec{\alpha }$ of the control regressors are fixed at their baseline model estimates when fitting models that include SNPs. The use of these estimates leads to great computational savings because of two reasons: first, the numerical optimization methods used for estimating $\kappa$ account for a substantial part of the computational cost of fitting LMMs; second, fixing $\varvec{\alpha }$ allows us to use fast numerical linear algebra to simultaneously estimate the regression coefficients of the SNPs in all LMMs that have just one SNP.

Specifically, we estimate $\kappa$ and $\varvec{\alpha }$ from the baseline model

2 $$\begin{aligned} {{\textbf {Y}}} \sim N(X_c \varvec{\alpha }, \sigma ^2(I + \kappa ZKZ^\top )). \end{aligned}$$

These estimates are calculated using the restricted likelihood (REML) which is equivalent to using a flat prior on $\varvec{\alpha }$, integrating out $\varvec{\alpha }$, and maximizing the corresponding integrated likelihood with respect to $\sigma ^2$ and $\kappa$. We then take an Empirical Bayes approach that assumes $\kappa$ and $\varvec{\alpha }$ are known parameters equal to their estimates $\hat{\kappa }$ and $\hat{\varvec{\alpha }}$. Let $\widetilde{{{\textbf {Y}}}} = {{\textbf {Y}}} - X_c \hat{\varvec{\alpha }}$, be the vector of residuals from the baseline model. Similar to SMA, the screening step estimates the regression coefficient $\beta _j$ of SNP *j*, $j = 1,\dots ,p$, in the approximate LMM

3 $$\begin{aligned} \widetilde{{{\textbf {Y}}}} {\mathop {\sim }\limits ^{a}} N(x_j \beta _j,\sigma _j^2(I + \hat{\kappa } ZKZ^\top )), \end{aligned}$$

where $x_j$ denotes the covariate related to the *j*th SNP. Let $\hat{\beta }_j = \left( x_j^\top (I + \hat{\kappa }ZKZ^\top )^{-1}x_j\right) ^{-1} x_j^\top (I + \hat{\kappa }ZKZ^\top )^{-1}\widetilde{{{\textbf {Y}}}}$ be the REML of $\beta _j$ under Eq.. (3). Then $\hat{\beta }_j|\beta _j {\mathop {\sim }\limits ^{a}} N(\beta _j,\sigma _{\beta _j}^2)$, where $\sigma _{\beta _j}^2 = \hat{\sigma }_j^2(x_j^\top (I + \hat{\kappa }ZKZ^\top )^{-1}x_j)^{-1}$. Note that $\hat{\sigma }_j^2$ is the REML estimate calculated for the model given by Eq. (3) for SNP *j*.

We assume a spike and slab prior for $\beta _j$ [19]. Traditionally, such a prior usually assumes for $\beta _j$ a mixture of a Dirac delta function and a Gaussian distribution [13, 19]. In contrast, instead of a Gaussian distribution, we assume a nonlocal prior which has better theoretical properties with respect to the convergence rates of posterior probabilities [9, 10]. Specifically, we assume that *a priori* $\beta _j$ follows a mixture of a Dirac delta prior and a moment nonlocal prior [9] of the form

4 $$\begin{aligned} p(\beta _j|\tau ,\pi _0) &= \pi _0 \delta (\beta _j = 0) + (1 - \pi _0) \frac{\beta _j^2 (x_j^\top (I + \hat{\kappa }ZKZ^\top )^{-1} x_j)}{n\tau \sigma _j^2} \\ &\quad \times N\left( \beta _j\mid 0,\frac{n\tau \sigma _j^2}{(x_j^\top (I + \hat{\kappa }ZKZ^\top )^{-1} x_j)}\right) . \end{aligned}$$

We note that in Eq. (4), we take a hierarchical modeling approach where the regression coefficients of all SNPs share the same parameters $\pi _0$ and $\tau$. We consider three different procedures for choosing $\tau$: fix $\tau = 0.348$ as recommended in [10]; fix $\tau = 0.022$ as recommended in [17]; or estimate $\tau$ from the data [11]. Finally, BGWAS borrows strength across SNPs by estimating either $\pi _0$ or $(\pi _0,\tau )$ in a computationally efficient Empirical Bayes approach.

We now discuss how to estimate $\pi _0$ and $\tau$. We assign a noninformative uniform prior on the interval (0,1) for the probability of a true null SNP $\pi _0$. As the uniform prior is bounded on the interval (0,1), this is a proper prior for $\pi _0$. For the scale parameter $\tau$, we assign an Inverse Gamma prior as proposed in [11] for generalized linear models. To set the hyperparameters of this Inverse Gamma prior, we note that [17] proposed to fix $\tau$ at 0.022 for GWAS analysis. Thus, we set the prior mean of $\tau$ to 0.022. In addition, we note that values of $\tau$ smaller than 0.01 would allow the selection of too many false SNPs. Further, values of $\tau$ that are too close to zero lead to numerical instabilities. Based on these considerations, we assign an Inverse Gamma prior with shape $0.55/0.022 + 1$ and scale 0.55 implying a prior mean of $\tau$ equal to 0.022. In addition, this choice implies the prior probability that $\tau$ is less than 0.01 is less than 0.001, stochastically bounding $\tau$ away from zero to make computations stable. As the simulation study in the Results Section shows, this choice of priors works very well for GWAS analysis.

Multiplying the corresponding density for $\hat{\beta }_j$ by the prior for $\beta _j$ given in Eq. (4) and integrating out $\beta _j$, we obtain the predictive density:

5 $$\begin{aligned} p(\hat{\beta }_j|\tau ,\pi _0) &= \int N(\hat{\beta }_j|\beta _j,\sigma ^2_{\beta _j}) p(\beta _j|\tau ,\pi _0)d\beta _j \\ & = \pi _0 N(\hat{\beta }_j\mid 0,\sigma ^2_{\beta _j}) + (1 - \pi _0)(2\pi \sigma ^2_{\beta _j})^{-1/2}(n\tau + 1)^{-3/2} \\ &\quad \times \left( 1 + \frac{n\tau \hat{\beta }^2_j}{(n\tau + 1)\sigma ^2_{\beta _j}} \right) \exp \left[ -\frac{\hat{\beta }^2_j}{2(n\tau + 1)\sigma ^2_{\beta _j}}\right] . \end{aligned}$$

The derivation of the predictive density is provided in the Additional file 1. Assuming that $\hat{\beta }_1,\hat{\beta }_2,\dots ,\hat{\beta }_p$ conditional on $\beta _1, \beta _2, \dots , \beta _p$ are approximately independent, an approximate likelihood function for $\pi _0$ and $\tau$ is given by

6 $$\begin{aligned} {\mathcal {L}}(\tau ,\pi _0;\hat{\beta }_1,\dots ,\hat{\beta }_p) = \prod _{j = 1}^p p(\hat{\beta }_j | \tau , \pi _0). \end{aligned}$$

Let $p(\tau )$ and $p(\pi _0)$ be the prior densities for $\tau$ and $\pi _0$, respectively. Then, by Bayes Theorem the joint posterior density of $\tau$ and $\pi _0$ is

7 $$\begin{aligned} p(\tau ,\pi _0| \hat{\beta }_1, \dots , \hat{\beta }_p) {\mathop {\propto }\limits ^{a}} {\mathcal {L}}(\tau ,\pi _0;\hat{\beta }_1,\dots ,\hat{\beta }_p) p(\tau ) p(\pi _0). \end{aligned}$$

BGWAS estimates $\pi _0$ and $\tau$ by maximizing the posterior density given in (7). When $\tau$ is treated as fixed, only $\pi _0$ is estimated from the posterior distribution. After that, BGWAS takes an Empirical Bayes approach that fixes $\pi _0 = \hat{\pi }_0$ and $\tau = \hat{\tau }$ to calculate the posterior probability of $\beta _j = 0$ for all *j* using the predictive density of $\hat{\beta }_j$. Specifically, applying Bayes theorem, the posterior probability is given by:

8 $$\begin{aligned} P(\beta _j = 0| \hat{\beta }_j,\hat{\tau },\hat{\pi }_0) = \frac{\hat{\pi }_0N(\hat{\beta }_j|0,\sigma ^2_{\beta _j})}{p(\hat{\beta }_j|\hat{\tau },\hat{\pi }_0)}. \end{aligned}$$

With the posterior probabilities of $\beta _j = 0$ for all SNPs, the BGWAS screening step uses a Bayesian FDR control procedure [5–8] to select a set of candidate SNPs. Let *k* be the number of candidate SNPs and $X_k$ be the design matrix that includes all such candidate SNPs.

### BGWAS model selection step

The BGWAS model selection step searches through the model space of all LMMs that contain any number of candidate SNPs in $X_k$. Similarly to the screening step, to speed up computations the model selection step uses estimates of $\kappa$ and $\varvec{\alpha }$ from a baseline model. Specifically, first $\kappa$ and $\varvec{\alpha }$ are estimated assuming as baseline model the full model

9 $$\begin{aligned} {{\textbf {Y}}} \sim N(X_c \varvec{\alpha }+ X_k \varvec{\beta }_k,\sigma ^2(I + \kappa ZKZ^\top )). \end{aligned}$$

These estimates are calculated using restricted maximum likelihood (REML) estimation. After that, for all other models we assume that $\kappa$ and $\varvec{\alpha }$ are known parameters equal to their estimates $\hat{\kappa }$ and $\hat{\varvec{\alpha }}$. Next, similarly to the screening step, define $\widetilde{{{\textbf {Y}}}} = {{\textbf {Y}}} - X_c \hat{\varvec{\alpha }}$. Now consider a model $M_l$ with *s* possible SNPs, where $0 \le s \le k$. Let $\varvec{\beta }_l$ be the vector of coefficients and $X_l$ be the covariate matrix associated with these *s* SNPs. Then, the distribution of $\widetilde{{{\textbf {Y}}}}$ in model $M_l$ is approximately

10 $$\begin{aligned} \widetilde{{{\textbf {Y}}}}|M_l {\mathop {\sim }\limits ^{a}} N(X_l \varvec{\beta }_l,\sigma _l^2(I + \hat{\kappa } ZKZ^\top )). \end{aligned}$$

We propose a novel product moment (pMOM) prior for Gaussian linear mixed models. This prior uses the Fisher Information matrix in its Gaussian kernel. We note that [11] proposed to use the *diagonal* of the Fisher Information matrix in the Gaussian kernel of a pMOM prior for generalized linear models. Instead of the diagonal of the Fisher Information matrix, our use of the full Fisher Information matrix allows for the high correlations among SNPs to be accounted for in the pMOM prior. Specifically, the prior we propose is

11 $$\begin{aligned} \pi (\varvec{\beta }_l|\hat{\tau },\hat{\sigma }_l^2) = d_l \prod _{i = 1}^s \beta _{li}^{2} \times N\left( \varvec{\beta }_l\mid 0, \hat{\tau } \hat{\sigma }_l^2 n (X_l^\top (I + \hat{\kappa }ZKZ^\top )^{-1}X_l)^{-1} \right) \end{aligned}$$

where

12 $$\begin{aligned} d_l = \left\{ \int _{{\mathbb {R}}^s} \prod _{i = 1}^s \beta _{li}^{2} \times N\left( \varvec{\beta }_l\mid 0, \hat{\tau } \hat{\sigma }_l^2 n (X_l^\top (I + \hat{\kappa }ZKZ^\top )^{-1}X_l)^{-1} \right) d\varvec{\beta }_l\right\} ^{-1}. \end{aligned}$$

Note that $\hat{\tau }$ is either estimated in the screening step or fixed at the chosen value.

The marginal likelihood $m_l(\widetilde{{{\textbf {Y}}}})$ is then

13 $$\begin{aligned} m_l(\widetilde{{{\textbf {Y}}}})= (2\pi \hat{\sigma }_l^2)^{-(\frac{n}{2})}|I + \hat{\kappa }ZKZ^\top |^{-1/2} (n\hat{\tau } + 1)^{-s/2} \exp \left( -\frac{R_l}{2\hat{\sigma }_l^2} \right) \frac{E_2(\prod _{i = 1}^s \beta _{li}^{2})}{E_1(\prod _{i = 1}^s \beta _{li}^{2})}, \end{aligned}$$

where

$$\begin{aligned} \begin{aligned} C_l&= \frac{n\hat{\tau } + 1}{n\hat{\tau }}X_l^\top (I + \hat{\kappa }ZKZ^\top )^{-1}X_l, \\ {\tilde{\varvec{\beta }}}_l&= C_l^{-1}X_l^\top (I + \hat{\kappa }ZKZ^\top )^{-1}\widetilde{{{\textbf {Y}}}}, \\ R_l&= \widetilde{{{\textbf {Y}}}}^\top (I + \hat{\kappa }ZKZ^\top )^{-1} [(I + \hat{\kappa }ZKZ^\top ) - X_l C_l^{-1}X_l^\top ](I + \hat{\kappa }ZKZ^\top )^{-1}\widetilde{{{\textbf {Y}}}}. \end{aligned} \end{aligned}$$

Here, $E_1(\prod _{i = 1}^s \beta _{li}^{2})$ is the expected value of $\prod _{i = 1}^s \beta _{li}^{2}$ with respect to $N(\varvec{\beta }_l|0,\hat{\sigma }_l^2 (n\hat{\tau } + 1) C_l^{-1})$ and $E_2(\prod _{i = 1}^s \beta _{li}^{2})$ is the expected value of $\prod _{i = 1}^s \beta _{li}^{2}$ with respect to $N(\varvec{\beta }_l|{\tilde{\varvec{\beta }}}_s,\hat{\sigma }_l^2 C_l^{-1})$. To compute both expectations, a Monte Carlo simulation obtains 1000 draws from the distribution $N(\varvec{\beta }_l|{\tilde{\varvec{\beta }}}_s,\hat{\sigma }_l^2 C_l^{-1})$ and performs a transformation of variables to get a second set of 1000 draws from $N(\varvec{\beta }_l|0,\hat{\sigma }_l^2 (n\hat{\tau } + 1) C_l^{-1})$. Now, these draws can be used to obtain Monte Carlo estimates of $E_1(\prod _{i = 1}^s \beta _{li}^{2})$ and $E_2(\prod _{i = 1}^s \beta _{li}^{2})$. Proof of the marginal likelihood derivation given in Eq. (13) is provided in the Additional file 1.

To assign the prior probability on a model $M_l$ with *s* SNPs, we assume that SNPs are true positives or true negatives according to a sequence of exchangeable Bernoulli trials with probability of a true negative equal to $\pi _0$. Thus, the prior probability of model $M_l$ with *s* SNPs is

14 $$\begin{aligned} p(M_l) = (\pi _0)^{(k - s)}(1 - \pi _0)^{s}. \end{aligned}$$

BGWAS implements this prior probability by setting $\pi _0$ equal to the estimated proportion $\hat{\pi }_0$ of true null SNPs estimated in the screening step.

Then, by Bayes Theorem the posterior probability of model $M_l$ is

15 $$\begin{aligned} P(M_l|\widetilde{{{\textbf {Y}}}}) = \frac{p(M_l)m_l(\widetilde{{{\textbf {Y}}}})}{\sum _{j = 1}^m p(M_j)m_j(\widetilde{{{\textbf {Y}}}})} \propto p(M_l)m_l(\widetilde{{{\textbf {Y}}}}), \end{aligned}$$

where $m = 2^k$ is the total number of possible models.

To perform model selection with the candidate SNPs identified in the screening step, BGWAS either uses complete enumeration (when the number of candidate SNPs is less than 16) or searches the model space with a genetic algorithm. Specifically, we have implemented a genetic algorithm with the function ga() from the R package GA [20] that iterates mutation, crossover, and selection steps.

Our implementation starts with an initial population of 100 models that includes one model with just the intercept and 99 models with only one SNP per model. If the screening step yields more than 99 candidate SNPs, then the 99 SNPs with the highest posterior probabilities are used in the initial population. If the screening step yields less than 99 candidate SNPs, then the remaining models in the initial population are chosen based on the GA package’s default settings. The fitness function used in this genetic algorithm is $\log (P(M_l)) + \log (m_l(\widetilde{{{\textbf {Y}}}}))$. The algorithm stops if either 4000 maximum iterations are reached or if convergence is achieved with 400 consecutive iterations having the same best model.

## Results

### Simulation study

To assess the performance of BGWAS compared to SMA, data have been simulated under the mixed effects model:

16 $$\begin{aligned} {{\textbf {Y}}} = \alpha {{\textbf {1}}} + X \varvec{\beta }+ Z {{\textbf {u}}} + \varvec{\epsilon }, \end{aligned}$$

where ${{\textbf {u}}} \sim N(0,\sigma ^2\kappa K)$ and $\varvec{\epsilon }\sim N(0,\sigma ^2 I)$. For this simulation study we consider two SMA procedures with Bonferroni correction: “SMA-Approx.” estimates variance components estimated from a baseline model [3, 18], “SMA-Exact” estimates variance components for each model [2, 21]. In addition, we consider BGWAS with the three different methods for the nonlocal prior procedure discussed in the BGWAS Screening Step section. These three nonlocal prior procedures differ in the way they specify the hyperparameter $\tau$: fix $\tau = 0.348$ as recommended in [10]; fix $\tau = 0.022$ as recommended in [17]; and estimate $\tau$ from the data assuming an Inverse Gamma prior with shape $0.55/0.022 + 1$ and scale 0.55. In all nonlocal prior based methods, we assume a uniform prior on the interval (0,1) for $\pi _0$. As discussed in [10] and [17], the fixed values of $\tau = 0.348$ or $\tau = 0.022$ assign 0.99 marginal prior probability to $|\beta _i| \ge 0.2\sigma$ or $|\beta _i| \ge 0.05\sigma$ respectively. As a consequence, pre-specifying different values of $\tau$ may have a large effect on the false discovery rates and true positive rates of nonlocal prior methods. As an alternative, estimating $\tau$ provides a data-driven way to set the scale parameter.

To assess performance of these methods we use three different criteria: number of true positives, number of false positives, and the F1 score. The F1 score is the harmonic mean of precision (one minus the false discovery rate) and recall (the number of detected true SNPs divided by the total number of true SNPs). Similarly to [22], we define true positives and false positives using a buffer region. Specifically, if one or more selected SNPs are in a 5 kilobase pair (kbp) window centered at a true causal SNP, then that is counted as one true positive. Selected SNPs not located in any of the true-causal-SNP buffer regions are declared false positives. This buffer region mirrors the way scientists decide to further investigate genes that are near SNPs identified in GWAS studies [23, 24].

This simulation study is rather extensive and the full results are shown in the Additional file 1. We consider four different sizes of genotype data, all subsetted from an Illumina sequencing of 2772 humans. The four different sizes reflect all considered combinations of two sample sizes ($n = 400$ and $n = 2772$) and number of SNPs ($p = 225{,}000$ and $p = 800{,}000$). When there are 225,000 SNPs, there are 15 causal SNPs starting at position 7500 and with 15,000 SNPs in between each causal SNP. When there are 800,000 SNPs there are 20 causal SNPs starting at position 20,000 and with 40,000 SNPs in between each causal SNP. We explore four different vectors of regression coefficients for each set of causal SNPs. The first vector of regression coefficients is a vector of zeros, that is, there are no causal SNPs. In the three other vectors of regression coefficients, all coefficients are equal to 0.4 except for the coefficients at positions 1, 5, 9, 13, and 17. At these positions ($\beta ^{(1)}$), the coefficients are equal to each other and take on the values of 0.1, 0.4, and 1.6 for each of the three vectors of regression coefficients. Further, we set $\sigma ^2 = 0.2$ and have three different values of $\kappa$: 0, 0.1, and 1. Note that when $\kappa = 0$ the true model does not have kinship random effects. In that case of $\kappa = 0$, we implement SMA with simple linear regression. However, note that in this simulation study we always implement BGWAS with LMMs. Finally, we illustrate this procedure using two different nominal FDR levels, the traditional 0.05 and a less conservative 0.1.

The remainder of this section is divided into three subsections: General Simulation Study examines the two combinations of parameters closest to the case studies; Behavior of BGWAS when there is no Kinship Dependence Structure investigates how well BGWAS with the nonlocal prior performs when there are no causal SNPs, that is, when all regression coefficients are 0; Behavior of BGWAS when there are no Causal SNPs investigates how BGWAS performs when data have been simulated from a linear model instead of a linear mixed model; and Recommendation provides a recommendation for which BGWAS procedure to use.

#### General simulation study

Here we focus on results of the simulation study for combinations of sample size, number of SNPs, and parameter values that best match the case studies we explore later in the Case Studies section. The first simulation study combination shown in Table 1 is similar to the *A. Thaliana* case study. The *A. Thaliana* case study has 328 observations and about 230,000 SNPs. Estimates from the best model suggest the closest simulation study combination of parameter values is $\kappa = 1$ and $\sigma ^2 = 0.2$. The closest matching set of coefficients is the third setting with positions 1, 5, 9, and 13 all taking the value 1.6. However, we show results for all three different settings of the coefficients for the two nominal FDR levels. The second simulation study combination shown in Table 2 is similar to the alcohol dependence case study, which considers the log of age of first drink with 1738 subjects and approximately 840,000 SNPs. The closest simulation study combination has $\kappa = 0.1$ and $\sigma ^2 = 0.2$ with the regression coefficients all equal to 0.4. For a full understanding of how each method performs in each combination, the Additional file 1 provides tables with the same information as shown in Tables 1 and 2 for all other combinations of sample size, number of SNPs, and parameter values. Tables 1 and 2 display results averaged over 50 datasets for each setting. The average number of true positives (TP), average number of false positives (FP), and average F1 score are given for each method for each setting. The best result for each nominal FDR in each column appears in boldface.

**Table 1:** Results for GWAS data simulated from LMM with $n = 400, p = 225{,}000, \kappa = 1$, and $\sigma ^2 = 0.2$

Nominal FDR	Method	$\beta ^{(1)} = 0.1$				$\beta ^{(1)} = 0.4$				$\beta ^{(1)} = 1.6$			
		TP	FP	F1	Time (s)	TP	FP	F1	Time (s)	TP	FP	F1	Time (s)
0.05	SMA-Approx.	5.2	6.9	0.38	**4**	4.6	4.1	0.39	**4**	3.9	36.4	0.14	**4**
	SMA-Exact	5.2	7.0	0.38	103	4.6	4.2	0.39	104	3.9	36.9	0.14	93
	NP, $\tau = 0.348$	4.2	**0**.**6**	0.42	37	3.1	**0**.**1**	0.34	17	4.0	**0**.**0**	0.42	29
	NP, $\tau = 0.022$	6.1	0.8	0.55	35	6.3	0.6	0.57	30	**4**.**1**	**0**.**0**	**0**.**43**	35
	NP, $\tau$ estimated	**6**.**4**	0.8	**0**.**57**	40	**6**.**7**	0.8	**0**.**60**	36	**4**.**1**	**0**.**0**	**0**.**43**	37
0.1	SMA-Approx.	5.5	8.1	0.39	**4**	5.3	5.2	0.42	**4**	4.0	41.4	0.13	**4**
	SMA-Exact	5.6	8.2	0.39	103	5.3	5.3	0.42	104	4.0	41.9	0.13	93
	NP, $\tau = 0.348$	4.6	**0**.**7**	0.45	32	3.9	**0**.**2**	0.40	33	4.0	**0**.**0**	0.42	31
	NP, $\tau = 0.022$	6.4	0.9	0.57	39	6.8	0.8	0.60	35	**4**.**4**	**0**.**0**	**0**.**45**	37
	NP, $\tau$ estimated	**6**.**6**	0.9	**0**.**59**	48	**7**.**0**	1.1	**0**.**61**	45	**4**.**4**	**0**.**0**	**0**.**45**	40

In this table, there are 15 causal SNPs. The regression coefficients of the 15 causal SNPs are $\varvec{\beta }= (\beta ^{(1)}, 0.4, 0.4, 0.4, \beta ^{(1)}, 0.4, 0.4, 0.4, \beta ^{(1)}, 0.4, 0.4, 0.4, \beta ^{(1)}, 0.4, 0.4)^\top$. TP indicates Average number of True Positives, FP is Average number of False Positives, and F1 is the Average F1 score. Average Performance of each method over 50 datasets for each setting

**Table 2:** Results for GWAS data simulated from LMM with $n = 2772, p = 800{,}000, \kappa = 0.1$, and $\sigma ^2 = 0.1$

Nominal FDR	Method	$\beta ^{(1)} = 0.1$				$\beta ^{(1)} = 0.4$				$\beta ^{(1)} = 1.6$			
		TP	FP	F1	Time (s)	TP	FP	F1	Time (s)	TP	FP	F1	Time (s)
0.05	SMA-Approx.	**14**.**1**	169.5	0.14	**139**	**18**.**7**	223.9	0.14	**95**	9.0	260.2	0.06	**106**
	SMA-Exact	**14**.**1**	169.8	0.14	1137	**18**.**7**	223.9	0.14	1093	9.0	260.2	0.06	1968
	NP, $\tau = 0.348$	13.0	**1**.**1**	0.76	259	16.6	**2**.**2**	0.85	281	8.4	**0**.**7**	0.58	208
	NP, $\tau = 0.022$	13.8	1.6	0.78	279	16.9	2.4	**0**.**86**	321	10.1	1.7	0.64	242
	NP, $\tau$ estimated	14.0	1.6	**0**.**79**	283	16.8	2.7	0.85	339	**10**.**9**	1.5	**0**.**67**	254
0.1	SMA-Approx.	**14**.**2**	176.6	0.14	**139**	**18**.**8**	234.5	0.14	**95**	9.1	274.4	0.06	**106**
	SMA-Exact	**14**.**2**	177.0	0.14	1137	**18**.**8**	234.5	0.14	1093	9.1	274.4	0.06	1968
	NP, $\tau = 0.348$	13.1	**1**.**4**	0.76	265	16.9	**2**.**1**	**0**.**87**	293	8.4	**0**.**9**	0.58	210
	NP, $\tau = 0.022$	14.0	1.6	**0**.**79**	289	17.1	2.4	**0**.**87**	313	11.2	1.4	0.69	252
	NP, $\tau$ estimated	**14**.**2**	1.8	**0**.**79**	291	16.9	2.8	0.85	340	**11**.**7**	1.4	**0**.**71**	267

In this table, there are 20 causal SNPs. The regression coefficients of the 20 causal SNPs are $\varvec{\beta }= (\beta ^{(1)}, 0.4, 0.4, 0.4, \beta ^{(1)}, 0.4, 0.4, 0.4, \beta ^{(1)}, 0.4, 0.4, 0.4, \beta ^{(1)}, 0.4, 0.4, 0.4, \beta ^{(1)}, 0.4, 0.4, 0.4)^\top$. TP indicates Average number of True Positives, FP is Average number of False Positives, and F1 is the Average F1 score. Average Performance of each method over 50 datasets for each setting

In both tables, BGWAS with nonlocal priors better controls false discoveries while maintaining a level recall of true SNPs similar to that of SMA. In terms of overall performance, the F1 score is highest for BGWAS in every setting in every simulation setting. In Table 1, BGWAS either with using $\tau = 0.022$ or estimating $\tau$ detects a higher number of true positives compared to SMA in all settings and with all FDR nominal levels. In addition, when compared to SMA, BGWAS reduces the number of false positives by a factor of 10 or more. In Table 2, the number of true positives detected by BGWAS with $\tau = 0.022$ or estimating $\tau$ is similar to the number of true positives detected by SMA when using a type 1 nominal level of 0.1. Importantly, in Table 2, BGWAS reduces false positives by a factor of 100 or more. The reduction in false positives is credited to both the BGWAS screening step and the BGWAS model selection step. The BGWAS screening step is less conservative than SMA and the BGWAS model selection step better controls FDR.

Different ways to specify $\tau$ in our BGWAS approach offer their own benefits. The use of $\tau = 0.348$ provides the best false discovery control out of any method but also has the lowest true positive rate out of any method. Thus BGWAS with $\tau = 0.348$ is by far the most conservative method. Overall, BGWAS using $\tau = 0.022$ well balances the true positives and false discoveries in nearly all settings. Finally, BGWAS estimating $\tau$ performs the best in most circumstances in terms of true positive rate.

The differences in performance of BGWAS in Tables 1 and 2 are mainly due to differences in sample size *n*, variance parameter $\kappa$, and number of possible SNPs *p*. Tables 1 and 2 are useful because their conditions are similar to those of the two case studies, thus the results in these two tables inform us about the reliability of the case studies results. However, to understand the impact of sample size, variance parameter, and number of possible SNPs on the performance of BGWAS and SMA methods, we need to also examine Tables S1 through S9 in the Additional file 1. Comparison of all the tables leads to three main conclusions. First, we note that larger values of the variance parameter $\kappa$ lead to a decrease in the performance of both BGWAS and SMA. Second, the impact of increasing the number of possible SNPs *p* depends on the sample size. If the sample size is small $n=400$, then increasing *p* from 225,000 to 800,000 causes severe deterioration in performance of both BGWAS and SMA. However, if the sample size is moderate $n=2772$, then increasing *p* from 225,000 to 800,000 has little impact on the performance. Third, when the sample size increases, both BGWAS and SMA are able to detect a larger number of true causal SNPs. However, when the sample size increases, the number of false discoveries increases tremendously for SMA. As a result, when the sample size increases the performance of SMA in terms of F1 either remains about the same (when $p = 800{,}000$) or deteriorates (when $p = 225{,}000$)—this happens because the simulation study is based on real-life correlated SNPs. In contrast, when the sample size increases, the number of false discoveries remains well controlled by BGWAS. As a result, as the sample size increases, the performance of BGWAS in terms of F1 becomes even better.

A major consideration in the application of GWAS methods is the computational cost of the procedures. Tables 1 and 2 show for each procedure the average time in seconds averaged over 50 datasets. All timings in these tables and in the Additional file 1 are for computations performed on 100 cores of a 128 core AMD EPYC 7702 with 256 GB. All operations were implemented in the R statistical language built with OpenBLAS for optimized numerical linear algebra [25]. As BGWAS is a two-step procedure, it can not be faster than the screening step. However, since both its screening step and the model selection step approximate the variance from a baseline model, the times for BGWAS are much faster than a traditional SMA such as EMMA [2]. In these tables the timings range from 2 to 8 times faster for BGWAS. Therefore, BGWAS with different choices of $\tau$ are accurate procedures that maintain true positive rates while dramatically reducing the number of false positives in an efficient manner.

#### Behavior of BGWAS when there is no kinship dependence structure

To understand how the nonlocal prior procedure performs when data are simulated from a linear model, we simulate 50 datasets from the model

17 $$\begin{aligned} {{\textbf {Y}}} = \alpha {{\textbf {1}}} + X \varvec{\beta }+ \varvec{\epsilon }, \end{aligned}$$

where $\varvec{\epsilon }\sim N(0,\sigma ^2 I)$. Similarly to the general simulation study, $\sigma ^2 = 0.2$. We use the four different combinations of data sizes as in the general simulation study, that is, $n = 400$ or $n = 2772$ and $p = 225{,}000$ or 800,000. We again use the same set of causal SNPs as in the general simulation study, where the number of causal SNPs is 15 or 20 with all positions having value of 0.4 besides positions 1, 5, 9, 13, and 17 where these positions take on the values of 0.1, 0.4, and 1.6. For the SMA procedure, we assume the linear model without the kinship random effect. Thus, the SMA procedure in this section is exact. Meanwhile, our BGWAS procedure assumes the LMM given in Eq. (17). However, note that for datasets where BGWAS estimates $\kappa$ to be 0, then BGWAS will behave as if the fitted model is a linear model with independent error structure.

**Table 3:** Results for GWAS data simulated from a linear model with $n = 400, p = 225{,}000$, and $\sigma ^2 = 0.2$

Nominal FDR	Method	$\beta ^{(1)} = 0.1$				$\beta ^{(1)} = 0.4$				$\beta ^{(1)} = 1.6$			
		TP	FP	F1	Time (s)	TP	FP	F1	Time (s)	TP	FP	F1	Time (s)
0.05	SMA-Exact	6.5	12.0	0.39	**19**	6.3	6.7	0.45	**19**	4.0	40.1	0.13	**19**
	NP, $\tau = 0.348$	6.0	**0**.**8**	0.55	31	5.7	**0**.**3**	0.54	46	4.0	**0**.**0**	0.42	31
	NP, $\tau = 0.022$	**7**.**2**	0.9	**0**.**62**	51	8.2	0.8	0.68	48	**4**.**1**	**0**.**0**	**0**.**43**	37
	NP, $\tau$ estimated	**7**.**2**	1.0	**0**.**62**	55	**8**.**5**	0.8	**0**.**70**	55	**4**.**1**	**0**.**0**	**0**.**43**	39
0.1	SMA-Exact	6.7	14.3	0.38	**19**	6.8	8.3	0.45	**19**	4.0	46.1	0.12	**19**
	NP, $\tau = 0.348$	6.3	**0**.**8**	0.57	33	6.3	**0**.**4**	0.58	28	4.0	**0**.**0**	0.42	32
	NP, $\tau = 0.022$	**7**.**5**	1.0	**0**.**64**	57	8.5	1.0	**0**.**69**	55	**4**.**4**	**0**.**0**	**0**.**45**	38
	NP, $\tau$ estimated	**7**.**5**	1.1	**0**.**64**	63	**8**.**6**	1.1	**0**.**69**	67	**4**.**4**	**0**.**0**	**0**.**45**	41

In this table, there are 15 causal SNPs. The regression coefficients of the 15 causal SNPs are $\varvec{\beta }= (\beta ^{(1)}, 0.4, 0.4, 0.4, \beta ^{(1)}, 0.4, 0.4, 0.4, \beta ^{(1)}, 0.4, 0.4, 0.4, \beta ^{(1)}, 0.4, 0.4)^\top$. TP indicates Average number of True Positives, FP is Average number of False Positives, and F1 is the Average F1 score. Average Performance of each method over 50 datasets for each setting

Table 3 presents average number of true positives, false positives, and F1 score for 50 simulated datasets for $n = 400$ and $p = 225{,}000$. The best result for each nominal FDR in each column appears in boldface. Table 3 has similar results to Table 1 in terms of true positives, false positives, and F1 score. Results for other combinations of sample size, number of SNPs, and parameter values for the case $\kappa = 0$ shown in full detail in the Additional file 1 are also similar to results for the linear mixed model. Therefore, BGWAS performs better than SMA even when the true model is a linear model without kinship random effects.

#### Behavior of BGWAS when there are no causal SNPs

To examine the behavior of BGWAS in the case when there is no true causal SNPs, we have simulated 50 datasets for each combination of sample sizes ($n = 400$ and $n = 2772$), number of SNPs ($p = 225{,}000$ and $p = 800{,}000$), and $\kappa$ ($\kappa = 0$, $\kappa = 0.1$, and $\kappa = 1$) from the model

18 $$\begin{aligned} {{\textbf {Y}}} = \alpha {{\textbf {1}}} + Z {{\textbf {u}}} + \varvec{\epsilon }, \end{aligned}$$

where ${{\textbf {u}}} \sim N(0,\sigma ^2\kappa K)$ and $\varvec{\epsilon }\sim N(0,\sigma ^2 I)$. Since there are no true causal SNPs in this simulation study, for each method we recorded the number of false positives. Table 4 presents the average number of false positives over the 50 datasets created under the several considered combinations of sample size, number of SNPs and $\kappa$. The best result for each nominal FDR in each column appears in boldface.

**Table 4:** Results for GWAS data simulated from either a LMM or linear model with no causal SNPs

Nominal FDR	Method	$n = 400$						$n = 2772$					
		$p = 225{,}000$			$p = 800{,}000$			$p = 225{,}000$			$p = 800{,}000$		
		$\kappa = 0$	$\kappa = 0.1$	$\kappa = 1$	$\kappa = 0$	$\kappa = 0.1$	$\kappa = 1$	$\kappa = 0$	$\kappa = 0.1$	$\kappa = 1$	$\kappa = 0$	$\kappa = 0.1$	$\kappa = 1$
0.05	SMA-Approx.	–	0.02	0.02	–	**0**.**00**	0.14	–	**0**.**00**	0.04	–	**0**.**00**	**0**.**00**
	SMA-Exact	0.02	0.02	0.02	0.02	**0**.**00**	0.14	0.12	**0**.**00**	0.04	0.12	**0**.**00**	**0**.**00**
	NP, $\tau = 0.348$	**0**.**00**	**0**.**00**	**0**.**00**	**0**.**00**	**0**.**00**	**0**.**00**	**0**.**00**	**0**.**00**	**0**.**00**	**0**.**00**	**0**.**00**	**0**.**00**
	NP, $\tau = 0.022$	0.02	0.02	0.02	0.04	0.04	0.08	0.02	**0**.**00**	**0**.**00**	0.02	**0**.**00**	**0**.**00**
	NP, $\tau$ estimated	0.02	**0**.**00**	0.02	0.02	0.02	0.02	**0**.**00**	**0**.**00**	**0**.**00**	**0**.**00**	**0**.**00**	**0**.**00**
0.1	SMA-Approx.	–	0.02	0.12	NA	0.02	0.20	–	0.04	0.08	–	0.06	**0**.**00**
	SMA-Exact	0.06	0.02	0.12	0.04	0.02	0.20	0.24	0.04	0.08	0.18	0.06	**0**.**00**
	NP, $\tau = 0.348$	**0**.**00**	**0**.**00**	**0**.**00**	**0**.**00**	**0**.**00**	**0**.**02**	**0**.**00**	**0**.**00**	**0**.**00**	**0**.**00**	**0**.**00**	**0**.**00**
	NP, $\tau = 0.022$	0.04	0.02	0.02	0.08	0.18	0.24	0.04	**0**.**00**	**0**.**00**	0.08	**0**.**00**	**0**.**00**
	NP, $\tau$ estimated	0.02	0.02	0.04	0.02	0.08	0.06	**0**.**00**	**0**.**00**	**0**.**00**	**0**.**00**	**0**.**00**	**0**.**00**

There is no approximate SMA when there is no kinship structure (i.e. $\kappa = 0$). Average Number of False Positives for each method over 50 datasets for each setting

All methods are relatively conservative when there are no causal SNPs. We note that keeping $\tau$ fixed at 0.348 is still the most conservative method out of all methods. More importantly, when there are no causal SNPs, BGWAS with an estimated $\tau$ from the data controls false discoveries better than fixing $\tau = 0.022$. Combined with the results from the general simulation study, BGWAS with estimating $\tau$ from the data is the best method.

#### Recommendation

We have considered BGWAS with multiple different choices of $\tau$ and different FDR nominal values. Both estimating $\tau$ from the data and setting $\tau$ at 0.022 have similar performance in terms of the F1 score in almost all the combinations of the simulation study parameters. We note that when $\tau$ is estimated, the prior is an Inverse Gamma prior with a prior mean of 0.022. Therefore, the similar performance of these two methods is expected. Estimating $\tau$ tends to have slightly higher false discoveries and slightly higher true positives comparatively in the general simulation study. In the case of no causal SNPs, when compared to fixing $\tau$ at 0.022, estimating $\tau$ had a smaller number of false discoveries. As the goal of GWAS is detection of true positive SNPs while maintaining false discoveries to a reasonable level, we think that estimating $\tau$ from the data is the best approach for conducting real GWAS analyses. In this same light, setting the nominal level at 0.1 instead of 0.05 provided similar F1 scores but higher number of true positives. Therefore, our recommendation for GWAS analyses is estimating $\tau$ from the data and using a FDR nominal level of 0.1.

### Case studies

To demonstrate the utility and flexibility of BGWAS, we present two case studies with real data analyses. First, BGWAS is applied to data from a published study of salt stress on the selfing species *A. Thaliana* [24]. Second, BGWAS is applied to a study of alcohol dependency in humans and explores the response variable “age of first drink”. To normalize and variance-stabilize the data, the logarithm transformation has been applied to age of first drink. To briefly highlight the differences between BGWAS and SMA, Table 5 presents the number of SNPs found by each method for each nominal FDR level.

**Table 5:** The number of SNPs identified by each method for each case study

Method	Salt stress		Age first drink	
	$\alpha$ = 0.05	$\alpha$ = 0.1	$\alpha$ = 0.05	$\alpha$ = 0.1
SMA-Approx.	22	25	8	8
SMA-Exact	22	26	8	8
NP, $\tau = 0.348$	4	4	1	2
NP, $\tau = 0.022$	5	8	3	4
NP, $\tau$ estimated	7	7	4	6

Multiple comparison corrections are based on the number of SNPs in a given genotype dataset

For each application and under each FDR level, BGWAS with different choices of $\tau$ yields a much smaller number of identified SNPs than the SMA procedures. In addition, the results of the simulation study suggest that many of the SNPs found by the SMA methods may be false positives. Therefore, following the recommendation from the early section, the remainder of this section discusses the SNPs discovered using BGWAS with estimating $\tau$ from the data.

#### Salt stress in *A. Thaliana*

We analyze data from a study that considers three different settings of salt stress to identify SNPs and their genes associated with the response of *A. Thaliana* to salt stress [24]. The three settings considered by [24] were a control setting, 75 mM of NaCl, and 125 mM of NaCl. Different measures of the root structure were taken to gauge how salt stress impacted the plants. In our case study, we analyze the average length of lateral root per main root length for 328 *A. Thaliana* accessions under 75 mM NaCl salt stress. Genotype data was sequenced in [26]. Only SNPs with minor allele frequency greater than 0.01 were included in the analysis.

Following the recommendation given earlier, here we discuss the SNPs found by BGWAS estimating $\tau$ from the data and with a nominal FDR level of 0.1. Of the seven SNPs identified, one SNP is perfectly correlated to two other SNPs and another SNP is perfectly correlated with another SNP, implying nine identified SNPs. The 9 SNPs are located in the genes AT1G48300.1, AT1G62500, nearby AT2G38970, AT3G60370, AT4G14305.1, AT4G39955, AT4G39970, AT4G40000, AT5G28500.1. SNPs found in AT4G39955, AT4G39970, and AT4G40000 are in linkage disequilibrium. Importantly, AT1G62500 (also known as DEG27) is a gene that becomes differentially expressed in the event of salt stress [27]. In addition, AT4G39955 is an $\alpha$/$\beta$-Hydrolases superfamily protein; these proteins have been shown to enhance salt tolerance in the sweet potato family [28].

#### Alcohol use disorder in humans

We consider publicly available data from The Collaborative Study on the Genetics of Alcoholism (COGA), which was performed to identify genetic factors associated with alcohol dependency [29]. In this case study we analyze the response variable “log of age of first drink” for 1738 people of European ancestry. Illumina sequencing provided approximately 1 million SNPs. Only SNPs with minor allele frequency larger than 0.01, not in the X/Y chromosomes, and with RS identifiers were investigated. To control for the effect of sex, this analysis was performed on the residuals of the linear mixed model for log age of first drink regressed on sex.

Following the recommendation given earlier, here we discuss the SNPs found by BGWAS estimating $\tau$ from the data and with a nominal FDR level of 0.1. The six SNPs discovered are located in genes KCNMA1, near PPIAP33, ANKS1B, RBL1, ABCF1, and LINC02237. We note that KCNMA1 is known as a gene associated with alcohol dependency [30]. In addition, in a study with people of Chinese Han ethnicity, ANKS1B has been associated with alcoholism [31]. Finally, genes RBL1 and ABCF1 may be good candidates for further investigation.

## Conclusion and future directions

We have proposed BGWAS, a novel Bayesian two-step procedure based on nonlocal priors for the analysis of GWAS data. In BGWAS, we propose in Eq. (4) a hierarchical approach where the regression coefficients for the several SNPs share the same mixing probability $\pi _0$ and the same scale parameter $\tau$. Thus, BGWAS borrows strength across SNPs to estimate $\pi _0$ and $\tau$ in a very efficient Empirical Bayes approach. With the estimates $\hat{\pi _0}$ and $\hat{\tau }$, in both screening and model selection steps, BGWAS uses Bayes theorem to efficiently compute posterior probabilities and make decisions on which SNPs to select. We note that it is not clear how to implement a classical/frequentist approach that would borrow strength across SNPs in a way similar to BGWAS. In addition, we note that our simulation studies with real SNP data show that, when compared to widely used frequentist procedures, BGWAS has favorable performance with much smaller FDR.

One important issue when using nonlocal priors is the specification of the scale parameter $\tau$. Previous literature has proposed $\tau =0.348$ for usual linear regression problems [10] and $\tau =0.022$ for GWAS analysis [17]. In contrast, here we propose an empirical Bayes procedure that estimates $\tau$ from the GWAS data. Our simulation studies show that, when compared to fixing $\tau$ at 0.348 or 0.022, our procedure that estimates $\tau$ performs the best in most circumstances in terms of true positive rates. In addition, in the case when there are no causal SNPs, our procedure that estimates $\tau$ from the data controls false discoveries better than fixing $\tau$ at 0.022. Therefore, our recommendation for GWAS analyses is to estimate $\tau$ from the data.

Of the nine SNPs found by BGWAS for the Salt Stress case study, two of the SNPs were found in genes that have associated salt stress publications. Given the results of the simulation setting most closely related to this case study, Table 1 in the manuscript, we strongly believe that most of the other SNPs found by BGWAS are worthy of further investigation. The human case study of AUD found six SNPs of which two were located in genes previously related to AUD in publications. Similarly to the *A. Thaliana* case study, the simulation setting most similar to that of the case study, Table 2 of the manuscript, suggests that the remainder of the SNPs found by BGWAS are highly likely to be true positives and worth further investigation.

There are many possible avenues for future research. For example, a potentially useful avenue is to extend our work to non-Gaussian data such as the number of lateral roots in plants or the indicator of alcohol dependency in studies of alcohol use disorder. Another possible area of research would be to extend BGWAS to BioBank scale data.

## Supplementary Information

**Additional file 1.** This file contains the derivation of the predictive density in the screening step, the derivation of the marginal density in the model selection step, and additional simulation study results.

## Data Availability

The A. Thaliana phenotype data and genotype data are available from the following sources: A. Thaliana phenotype data available at https://arapheno.1001genomes.org; A. Thaliana genotype dataset available from R package qtcat.data (https://rdrr.io/github/QTCAT/qtcat.data/. Genotype and phenotype data for alcohol use disorder in humans is available from the NIH dbGap website: https://www.ncbi.nlm.nih.gov/gap/, the accession number is phs000092.v1.p1.

## Abbreviations

SMA: Single marker analysis
LMMs: Linear mixed models
pMOM: Product moment
MCMC: Markov mhain Monte Carlo
REML: Restricted likelihood
GWAS: Genome-wide association studies
SNPs: Single nucleotide polymorphisms
BICOSS: Bayesian Iterative Conditional Stochastic Search
FDR: False discovery rate
GA: Genetic algorithm
BIC: Bayesian information criterion
MAF: Minor allele frequency
