# Supplementary material for: BGWAS: Bayesian variable selection in linear mixed models with nonlocal priors for genome-wide association studies
Source: BMC Bioinformatics. 2023 May 11;24:194. doi: 10.1186/s12859-023-05316-x (PMC10176706; doi:10.1186/s12859-023-05316-x)
Supplement: Supplementary file 1 — Additional file 1. This file contains the derivation of the predictive density in the screening step, the derivation of the marginal density in the model selection step, and additional simulation study results. [file 12859_2023_5316_MOESM1_ESM.pdf]

Supplementary Material for BGWAS: Bayesian variable selection in linear mixed models with nonlocal priors for genome-wide association studies

S1 Derivation of Predictive Density in Screening Step

Under the model given in Equation (2) of the manuscript, we provide the full derivation of the predictive density given in Equation (5) of the manuscript. Assume the approximate LMM given in Equation (3), the REML $\hat{\beta}_j$ based on Equation (3), and the prior for β_j given in Equation (4) of the manuscript. Then, the predictive density is

$$
\begin{aligned}
p(\hat{\beta}_j|\tau,\pi_0) &= \int_{\beta_j} p(\hat{\beta}_j|\beta_j)p(\beta_j|\tau,\pi_0)d\beta_j \\
&= \int_{\beta_j} N(\hat{\beta}_j \mid \beta_j, \sigma^2_{\beta_j}) \times \left[\pi_0\delta(\beta_j=0) + (1-\pi_0)\frac{\beta_j^2}{n\tau\sigma^2_{\beta_j}} N(\beta_j \mid 0, n\tau\sigma^2_{\beta_j})\right] d\beta_j \\
&= \pi_0 N(\hat{\beta}_j \mid 0, \sigma^2_{\beta_j}) + (1-\pi_0)(2\pi\sigma^2_{\beta_j})^{-1/2}(n\tau+1)^{-3/2}\left(1+\frac{n\tau\hat{\beta}_j^2}{(n\tau+1)\sigma^2_{\beta_j}}\right)\exp\left[-\frac{\hat{\beta}_j^2}{2(n\tau+1)\sigma^2_{\beta_j}}\right] \\
&\quad\times \int_{\beta_j} N(\hat{\beta}_j \mid 0, \sigma^2_{\beta_j})\frac{\beta_j^2}{n\tau\sigma^2_{\beta_j}} N(\beta_j \mid 0, n\tau\sigma^2_{\beta_j})d\beta_j \\
&= \pi_0 N(\hat{\beta}_j \mid 0, \sigma^2_{\beta_j}) + (1-\pi_0)(2\pi\sigma^2_{\beta_j})^{-1/2}(n\tau+1)^{-3/2}\left(1+\frac{n\tau\hat{\beta}_j^2}{(n\tau+1)\sigma^2_{\beta_j}}\right)\exp\left[-\frac{\hat{\beta}_j^2}{2(n\tau+1)\sigma^2_{\beta_j}}\right].
\end{aligned}
$$

S2 Derivation of Marginal Density in Model Selection Step

Assume the full model provided in Equation (9), the approximated distribution given in Equation (10), and the prior for $\boldsymbol{\beta}_l$ given in Equation (11). Then, the marginal likelihood $m(\tilde{\mathbf{Y}})$ for model M_l is:

$$
\begin{aligned}
m_l(\tilde{\mathbf{Y}}) &= \int_{\boldsymbol{\beta}_l} p(\tilde{\mathbf{Y}}|\boldsymbol{\beta}_l)\pi(\boldsymbol{\beta}_l|\hat{\tau},\hat{\sigma}_l^2)d\boldsymbol{\beta}_l \\
&= d_l(2\pi)^{-(\frac{n+s}{2})}\left|\hat{\sigma}_l^2(I+\hat{\kappa}ZKZ^\top)\right|^{-1/2}(\hat{\tau}\hat{\sigma}_l^2 n)^{-s-s/2}|X_l^\top(I+\hat{\kappa}ZKZ^\top)^{-1}X_l|^{3/2} \\
&\quad\times\int_{\boldsymbol{\beta}_l}\exp\left[-\frac{1}{2\hat{\sigma}_l^2}\left((\tilde{\mathbf{Y}}-X_l\boldsymbol{\beta}_l)^\top(I+\hat{\kappa}ZKZ^\top)^{-1}(\tilde{\mathbf{Y}}-X_l\boldsymbol{\beta}_l)+\boldsymbol{\beta}_l^\top\frac{X_l^\top(I+\hat{\kappa}ZKZ^\top)^{-1}X_l}{\hat{\tau}n}\boldsymbol{\beta}_l\right)\right]\prod_{i=1}^s\beta_{li}^2 d\boldsymbol{\beta}_l.
\end{aligned}
$$

Let $\Sigma = I + \hat{\kappa}ZKZ^\top$. Then

$$
(\tilde{\mathbf{Y}}-X_l\boldsymbol{\beta}_l)^\top\Sigma^{-1}(\tilde{\mathbf{Y}}-X_l\boldsymbol{\beta}_l)+\boldsymbol{\beta}_l^\top\frac{X_l^\top\Sigma^{-1}X_l}{\hat{\tau}n}\boldsymbol{\beta}_l = * \qquad (1)
$$

can be written as

$$
\begin{aligned}
* &= \tilde{\mathbf{Y}}^\top \Sigma^{-1}\tilde{\mathbf{Y}} - 2\boldsymbol{\beta}_l^\top X_l^\top \Sigma^{-1}\tilde{\mathbf{Y}} + \boldsymbol{\beta}_l^\top X_l \Sigma^{-1} X_l \boldsymbol{\beta}_l + \boldsymbol{\beta}_l \frac{X_l^\top \Sigma^{-1} X_l}{\hat{\tau} n}\boldsymbol{\beta}_l \\
&= \tilde{\mathbf{Y}}^\top \Sigma^{-1}\tilde{\mathbf{Y}} - 2\boldsymbol{\beta}_l^\top X_l^\top \Sigma^{-1}\tilde{\mathbf{Y}} + \boldsymbol{\beta}_l^\top X_l \Sigma^{-1} X_l \boldsymbol{\beta}_l + \boldsymbol{\beta}_l \frac{X_l^\top \Sigma^{-1} X_l}{\hat{\tau} n}\boldsymbol{\beta}_l \\
&\qquad + \tilde{\mathbf{Y}}^\top \Sigma^{-1} X_l \left(\frac{n\hat{\tau}+1}{n\hat{\tau}} X_l^\top \Sigma^{-1} X_l\right)^{-1} X_l^\top \Sigma^{-1}\tilde{\mathbf{Y}} - \tilde{\mathbf{Y}}^\top \Sigma^{-1} X_l \left(\frac{n\hat{\tau}+1}{n\hat{\tau}} X_l^\top \Sigma^{-1} X_l\right)^{-1} X_l^\top \Sigma^{-1}\tilde{\mathbf{Y}} \\
&= \tilde{\mathbf{Y}}^\top \Sigma^{-1}\left[\Sigma - X_l \left(\frac{n\hat{\tau}+1}{n\hat{\tau}} X_l^\top \Sigma^{-1} X_l\right)^{-1} X_l^\top\right]\Sigma^{-1}\tilde{\mathbf{Y}} \\
&\qquad + \left(\boldsymbol{\beta}_l - \left(\frac{n\hat{\tau}+1}{n\hat{\tau}} X_l^\top \Sigma^{-1} X_l\right)^{-1} X_l^\top \Sigma^{-1}\tilde{\mathbf{Y}}\right)\left(\frac{n\hat{\tau}+1}{n\hat{\tau}} X_l^\top \Sigma^{-1} X_l\right)\left(\boldsymbol{\beta}_l - \left(\frac{n\hat{\tau}+1}{n\hat{\tau}} X_l^\top \Sigma^{-1} X_l\right)^{-1} X_l^\top \Sigma^{-1}\tilde{\mathbf{Y}}\right).
\end{aligned}
$$

Let $C_l = \frac{n\hat{\tau}+1}{n\hat{\tau}} X_l^\top \Sigma^{-1} X_l$, $\tilde{\boldsymbol{\beta}}_l = C_l^{-1} X_l^\top \Sigma^{-1}\tilde{\mathbf{Y}}$, and $R_l = \tilde{\mathbf{Y}}^\top \Sigma^{-1}\left[\Sigma - X_l C_l^{-1} X_l^\top\right]\Sigma^{-1}\tilde{\mathbf{Y}}$. Then, the marginal likelihood can be written as

$$
\begin{aligned}
m_l(\tilde{\mathbf{Y}}) &= d_l (2\pi)^{-(\frac{n+s}{2})} |\hat{\sigma}_l^2 \Sigma|^{-1/2} (\hat{\tau}\hat{\sigma}_l^2 n)^{-p-p/2} |X_l^\top \Sigma^{-1} X_l|^{3/2} \\
&\qquad \times \int_{\boldsymbol{\beta}_l} \exp\left[-\frac{1}{2\hat{\sigma}_l^2}\left(R_l + (\boldsymbol{\beta}_l - \tilde{\boldsymbol{\beta}}_l)^\top C_l (\boldsymbol{\beta}_l - \tilde{\boldsymbol{\beta}}_l)\right)\right] \prod_{i=1}^s \beta_{li}^2 d\boldsymbol{\beta}_l \\
&= d_l (2\pi)^{-(\frac{n}{2})} |\hat{\sigma}_l^2 \Sigma|^{-1/2} (\hat{\tau}\hat{\sigma}_l^2 n)^{-s-s/2} |X_l^\top \Sigma^{-1} X_l|^{3/2} \exp\left[-\frac{1}{2\hat{\sigma}_l^2} R_l\right] |\hat{\sigma}_l^2 C_l^{-1}|^{1/2} \\
&\qquad \times \int_{\boldsymbol{\beta}_l} (2\pi)^{-s/2} |\hat{\sigma}_l^2 C_l^{-1}|^{-1/2} \exp\left[-\frac{1}{2\hat{\sigma}_l^2}(R_l + (\boldsymbol{\beta}_l - \tilde{\boldsymbol{\beta}}_l)^\top C_l (\boldsymbol{\beta}_l - \tilde{\boldsymbol{\beta}}_l))\right] \prod_{i=1}^s \beta_{li}^2 d\boldsymbol{\beta}_l \\
&= d_l (2\pi)^{-(\frac{n}{2})} |\Sigma|^{-1/2} (\hat{\tau} n)^{-s-s/2} |X_l^\top \Sigma^{-1} X_l|^{3/2} \exp\left[-\frac{1}{2\hat{\sigma}_l^2} R_l\right] (\hat{\sigma}_l^2)^{-s-n/2} \\
&\qquad \times |\frac{n\hat{\tau}}{n\hat{\tau}+1} (X_l^\top \Sigma^{-1} X_l)^{-1}|^{1/2} E_2\left(\prod_{i=1}^s \beta_{li}^2\right) \\
&= d_l (2\pi)^{-(\frac{n}{2})} |\Sigma|^{-1/2} (\hat{\tau} n)^{-s} (n\hat{\tau}+1)^{-s/2} |X_l^\top \Sigma^{-1} X_l|^{1} \exp\left[-\frac{1}{2\hat{\sigma}_l^2} R_l\right] (\hat{\sigma}_l^2)^{-s-n/2} \\
&\qquad \times E_2\left(\prod_{i=1}^s \beta_{li}^2\right),
\end{aligned}
$$

where $E_2\left(\prod_{i=1}^s \beta_{li}^2\right)$ is the expected value of $\prod_{i=1}^s \beta_{li}^2$ with respect to $N\left(\boldsymbol{\beta}_l | \tilde{\boldsymbol{\beta}}_l, \hat{\sigma}_l^2 C_l^{-1}\right)$.

In addition, the integration constant d_l can be written as

$$
\begin{aligned}
d_l &= \left[\int_{\boldsymbol{\beta}_l} (2\pi)^{-s/2}(\hat{\tau}n\hat{\sigma}_l^2)^{-s-s/2}|X_l^\top \Sigma^{-1} X_l|^{3/2} \exp\left[-\frac{1}{2\hat{\sigma}_l^2}\boldsymbol{\beta}_l^\top \frac{1}{n\hat{\tau}+1} C_l \boldsymbol{\beta}_l\right] \prod_{i=1}^{s} \beta_{li}^2 d\boldsymbol{\beta}_l\right]^{-1} \\
&= \Big[(\hat{\tau}n\hat{\sigma}_l^2)^{-s-s/2}|X_l^\top \Sigma^{-1} X_l|^{3/2}|\hat{\sigma}_l^2(n\hat{\tau}+1)C_l^{-1}|^{1/2} \\
&\qquad \times \int_{\boldsymbol{\beta}_l} (2\pi)^{-s/2}|\hat{\sigma}_l^2(n\hat{\tau}+1)C_l^{-1}|^{-1/2} \exp\left[-\frac{1}{2\hat{\sigma}_l^2}\boldsymbol{\beta}_l^\top \frac{1}{n\hat{\tau}+1} C_l \boldsymbol{\beta}_l\right] \prod_{i=1}^{s} \beta_{li}^2 d\boldsymbol{\beta}_l\Big]^{-1} \\
&= \left[(\hat{\tau}n\hat{\sigma}_l^2)^{-s-s/2}|X_l^\top \Sigma^{-1} X_l|^{3/2}|\hat{\sigma}_l^2(n\hat{\tau}+1)C_l^{-1}|^{1/2} \times E_1\left(\prod_{i=1}^{s} \beta_{li}^2\right)\right]^{-1} \\
&= \left[(\hat{\tau}n\hat{\sigma}_l^2)^{-s-s/2}|X_l^\top \Sigma^{-1} X_l|^{3/2} \left|\hat{\sigma}_l^2(n\hat{\tau}+1)\left(\frac{n\hat{\tau}}{n\hat{\tau}+1} X_l^\top \Sigma^{-1} X_l\right)\right|^{1/2} \times E_1\left(\prod_{i=1}^{s} \beta_{li}^2\right)\right]^{-1} \\
&= \left[(\hat{\sigma}_l^2)^{-s}(\hat{\tau}n)^{-s}|X_l^\top \Sigma^{-1} X_l|^{1} \times E_1\left(\prod_{i=1}^{s} \beta_{li}^2\right)\right]^{-1} \\
&= (\hat{\sigma}_l^2)^{s}(\hat{\tau}n)^{s}|X_l^\top \Sigma^{-1} X_l|^{-1} \times \left[E_1\left(\prod_{i=1}^{s} \beta_{li}^2\right)\right]^{-1},
\end{aligned}
$$

where $E_1\left(\prod_{i=1}^{s} \beta_{li}^2\right)$ is the expected value of $\prod_{i=1}^{s} \beta_{li}^2$ with respect to $N\left(\boldsymbol{\beta}_l|0, \hat{\sigma}_l^2(n\hat{\tau}+1)C_l^{-1}\right)$. Then the marginal likelihood $m_l(\tilde{\mathbf{Y}})$ can be written as

$$
\begin{aligned}
m_l(\tilde{\mathbf{Y}}) &= d_l (2\pi)^{-(\frac{n}{2})}|\Sigma|^{-1/2}(\hat{\tau}n)^{-s}(n\hat{\tau}+1)^{-s/2}|X_l^\top \Sigma^{-1} X_l|^{1} \exp\left[-\frac{1}{2\hat{\sigma}_l^2} R_l\right] (\hat{\sigma}_l^2)^{-s-n/2} E_2\left(\prod_{i=1}^{s} \beta_{li}^2\right) \\
&= (2\pi\hat{\sigma}_l^2)^{-(\frac{n}{2})}|\Sigma|^{-1/2}(n\hat{\tau}+1)^{-s/2} \exp\left[-\frac{R_l}{2\hat{\sigma}_l^2}\right] \frac{E_2\left(\prod_{i=1}^{s} \beta_{li}^2\right)}{E_1\left(\prod_{i=1}^{s} \beta_{li}^2\right)}.
\end{aligned}
$$

S3 Additional Simulation Study Results

This section presents additional simulation study results.

Table S1: Results for GWAS data simulated from LMM with $n = 400$, $p = 225,000$, $\kappa = 0.1$, and $\sigma^2 = 0.2$. In this table, there are 15 causal SNPs. The regression coefficients of the 15 causal SNPs are $\boldsymbol{\beta} = (\beta^{(1)}, 0.4, 0.4, 0.4, \beta^{(1)}, 0.4, 0.4, 0.4, \beta^{(1)}, 0.4, 0.4, 0.4, \beta^{(1)}, 0.4, 0.4)^\top$. TP indicates Average number of True Positives, FP is Average number of False Positives, and F1 is the Average F1 score. Average Performance of each method over 50 datasets for each setting.

Nominal FDR	Method	$\beta^{(1)} = 0.1$				$\beta^{(1)} = 0.4$				$\beta^{(1)} = 1.6$			
		TP	FP	F1	Time (s)	TP	FP	F1	Time (s)	TP	FP	F1	Time (s)
0.05	SMA-Approx.	6.4	10.2	0.41	4	6.0	6.4	0.44	4	4.0	39.1	0.14	4
	SMA-Exact	6.5	10.3	0.41	100	6.1	6.6	0.44	100	4.0	39.7	0.14	90
	NP, $\tau = 0.348$	6.1	0.5	0.56	28	5.4	0.3	0.52	42	4.0	0.0	0.42	30
	NP, $\tau = 0.022$	7.4	0.8	0.64	48	7.9	0.7	0.67	46	4.1	0.0	0.43	36
	NP, τ estimated	7.4	1.0	0.64	58	7.8	1.0	0.66	53	4.1	0.0	0.43	38
0.1	SMA-Approx.	6.7	11.3	0.41	4	6.6	7.9	0.45	4	4.0	44.7	0.13	4
	SMA-Exact	6.8	11.6	0.41	100	6.6	8.1	0.45	100	4.0	45.5	0.12	90
	NP, $\tau = 0.348$	6.6	0.4	0.60	32	5.9	0.4	0.55	35	4.0	0.0	0.42	31
	NP, $\tau = 0.022$	7.6	0.9	0.65	57	8.1	1.1	0.67	55	4.4	0.0	0.45	39
	NP, τ estimated	7.8	0.9	0.66	58	8.3	1.1	0.68	62	4.4	0.0	0.45	40

Table S2: Results for GWAS data simulated from a linear model with $n = 2,772$, $p = 225,000$, and $\sigma^2 = 0.2$. In this table, there are 15 causal SNPs. The regression coefficients of the 15 causal SNPs are $\boldsymbol{\beta} = (\beta^{(1)}, 0.4, 0.4, 0.4, \beta^{(1)}, 0.4, 0.4, 0.4, \beta^{(1)}, 0.4, 0.4, 0.4, \beta^{(1)}, 0.4, 0.4)^\top$. TP indicates Average number of True Positives, FP is Average number of False Positives, and F1 is the Average F1 score. Average Performance of each method over 50 datasets for each setting.

Nominal FDR	Method	$\beta^{(1)} = 0.1$				$\beta^{(1)} = 0.4$				$\beta^{(1)} = 1.6$			
		TP	FP	F1	Time (s)	TP	FP	F1	Time (s)	TP	FP	F1	Time (s)
0.05	SMA-Exact	11.1	201.5	0.10	34	14.6	247.8	0.11	33	12.4	222.1	0.10	33
	NP, $\tau = 0.348$	9.8	1.2	0.75	126	13.6	1.0	0.92	164	12.4	0.4	0.89	145
	NP, $\tau = 0.022$	10.3	1.9	0.75	142	13.8	1.4	0.91	205	12.5	0.5	0.89	151
	NP, τ estimated	10.3	2.3	0.75	154	13.4	2.0	0.88	212	12.4	0.6	0.88	163
0.1	SMA-Exact	11.2	210.6	0.09	34	14.8	262.1	0.10	33	12.8	227.9	0.10	33
	NP, $\tau = 0.348$	10.0	1.3	0.76	132	13.8	1.1	0.92	187	12.6	0.4	0.90	146
	NP, $\tau = 0.022$	10.4	2.2	0.76	149	13.6	1.7	0.90	213	12.4	0.6	0.89	156
	NP, τ estimated	10.6	2.5	0.75	162	13.5	1.9	0.89	223	12.1	0.9	0.87	165

Table S3: Results for GWAS data simulated from a LMM with $n = 2,772$, $p = 225,000$, $\kappa = 0.1$, and $\sigma^2 = 0.2$. In this table, there are 15 causal SNPs. The regression coefficients of the 15 causal SNPs are $\boldsymbol{\beta} = (\beta^{(1)}, 0.4, 0.4, 0.4, \beta^{(1)}, 0.4, 0.4, 0.4, \beta^{(1)}, 0.4, 0.4, 0.4, \beta^{(1)}, 0.4, 0.4)^\top$. TP indicates Average number of True Positives, FP is Average number of False Positives, and F1 is the Average F1 score. Average Performance of each method over 50 datasets for each setting.

Nominal FDR	Method	$\beta^{(1)} = 0.1$				$\beta^{(1)} = 0.4$				$\beta^{(1)} = 1.6$			
		TP	FP	F1	Time (s)	TP	FP	F1	Time (s)	TP	FP	F1	Time (s)
0.05	SMA-Approx.	10.9	183.8	0.10	26	14.1	221.0	0.11	26	12.3	218.8	0.10	26
	SMA-Exact	10.9	184.8	0.10	330	14.1	221.8	0.11	318	12.3	219.0	0.10	290
	NP, $\tau = 0.348$	9.6	1.3	0.74	124	13.4	0.9	0.91	162	12.5	0.3	0.90	140
	NP, $\tau = 0.022$	10.3	1.8	0.76	140	13.7	1.4	0.91	188	12.5	0.5	0.90	149
	NP, τ estimated	10.5	2.0	0.76	157	13.5	1.8	0.89	205	12.2	0.9	0.87	159
0.1	SMA-Approx.	11.0	194.4	0.10	26	14.3	234.5	0.11	26	12.7	223.4	0.10	26
	SMA-Exact	11.0	195.3	0.10	330	14.3	235.2	0.11	318	12.7	223.6	0.10	290
	NP, $\tau = 0.348$	9.9	1.3	0.76	128	13.6	0.9	0.92	158	12.6	0.4	0.90	141
	NP, $\tau = 0.022$	10.3	2.0	0.75	150	13.6	1.5	0.90	196	12.5	0.5	0.89	158
	NP, τ estimated	10.4	2.5	0.74	160	13.5	1.9	0.89	220	12.3	0.7	0.88	175

Table S4: Results for GWAS data simulated from a LMM with $n = 2,772$, $p = 225,000$, $\kappa = 1$, and $\sigma^2 = 0.2$. In this table, there are 15 causal SNPs. The regression coefficients of the 15 causal SNPs are $\boldsymbol{\beta} = (\beta^{(1)}, 0.4, 0.4, 0.4, \beta^{(1)}, 0.4, 0.4, 0.4, \beta^{(1)}, 0.4, 0.4, 0.4, \beta^{(1)}, 0.4, 0.4)^\top$. TP indicates Average number of True Positives, FP is Average number of False Positives, and F1 is the Average F1 score. Average Performance of each method over 50 datasets for each setting.

Nominal FDR	Method	$\beta^{(1)} = 0.1$				$\beta^{(1)} = 0.4$				$\beta^{(1)} = 1.6$			
		TP	FP	F1	Time (s)	TP	FP	F1	Time (s)	TP	FP	F1	Time (s)
0.05	SMA-Approx.	10.3	146.8	0.12	34	13.6	188.5	0.13	33	11.8	215.6	0.10	31
	SMA-Exact	10.3	147.4	0.12	333	13.6	189.1	0.12	321	11.8	215.7	0.10	331
	NP, $\tau = 0.348$	9.1	1.1	0.73	124	12.8	0.9	0.89	155	11.6	0.7	0.85	142
	NP, $\tau = 0.022$	9.7	1.7	0.73	141	13.0	1.9	0.87	187	12.4	0.7	0.88	161
	NP, τ estimated	9.8	2.4	0.72	145	12.9	2.6	0.84	201	12.2	0.8	0.87	159
0.1	SMA-Approx.	10.5	156.2	0.12	34	13.7	200.8	0.12	33	12.4	221.1	0.10	31
	SMA-Exact	10.5	156.6	0.12	333	13.7	201.2	0.12	321	12.4	221.3	0.10	331
	NP, $\tau = 0.348$	9.2	1.2	0.72	123	12.8	1.1	0.88	158	12.1	0.8	0.87	145
	NP, $\tau = 0.022$	9.9	1.8	0.74	140	12.9	2.1	0.86	194	12.0	1.0	0.86	159
	NP, τ estimated	10.0	2.6	0.73	154	12.8	3.0	0.83	204	12.2	0.8	0.87	169

Table S5: Results for GWAS data simulated from a linear model with $n = 400$, $p = 800,000$, and $\sigma^2 = 0.2$. In this table, there are 20 causal SNPs. The regression coefficients of the 20 causal SNPs are $\boldsymbol{\beta} = (\beta^{(1)}, 0.4, 0.4, 0.4, \beta^{(1)}, 0.4, 0.4, 0.4, \beta^{(1)}, 0.4, 0.4, 0.4, \beta^{(1)}, 0.4, 0.4, 0.4, \beta^{(1)}, 0.4, 0.4, 0.4)^\top$. TP indicates Average number of True Positives, FP is Average number of False Positives, and F1 is the Average F1 score. Average Performance of each method over 50 datasets for each setting.

Nominal FDR	Method	$\beta^{(1)} = 0.1$				$\beta^{(1)} = 0.4$				$\beta^{(1)} = 1.6$			
		TP	FP	F1	Time (s)	TP	FP	F1	Time (s)	TP	FP	F1	Time (s)
0.05	SMA-Exact	2.1	1.2	0.18	71	1.5	0.4	0.14	66	5.0	46.3	0.14	71
	NP, $\tau = 0.348$	1.8	0.0	0.16	13	1.0	0.0	0.09	13	4.4	0.6	0.35	58
	NP, $\tau = 0.022$	3.3	0.2	0.28	18	2.2	0.0	0.19	15	4.4	0.6	0.35	63
	NP, τ estimated	3.4	0.2	0.29	33	2.3	0.1	0.20	27	4.1	0.9	0.33	65
0.1	SMA-Exact	2.5	1.5	0.20	71	1.8	0.4	0.16	66	5.0	49.6	0.13	71
	NP, $\tau = 0.348$	2.1	0.1	0.19	13	1.3	0.0	0.12	13	4.4	0.6	0.35	56
	NP, $\tau = 0.022$	3.6	0.3	0.30	27	2.6	0.1	0.23	21	4.3	0.7	0.34	62
	NP, τ estimated	3.9	0.4	0.32	40	2.6	0.2	0.22	27	4.2	0.8	0.34	68

Table S6: Results for GWAS data simulated from a LMM with $n = 400$, $p = 800,000$, $\kappa = 0.1$, and $\sigma^2 = 0.2$. In this table, there are 20 causal SNPs. The regression coefficients of the 20 causal SNPs are $\boldsymbol{\beta} = (\beta^{(1)}, 0.4, 0.4, 0.4, \beta^{(1)}, 0.4, 0.4, 0.4, \beta^{(1)}, 0.4, 0.4, 0.4, \beta^{(1)}, 0.4, 0.4, 0.4, \beta^{(1)}, 0.4, 0.4, 0.4)^\top$. TP indicates Average number of True Positives, FP is Average number of False Positives, and F1 is the Average F1 score. Average Performance of each method over 50 datasets for each setting.

Nominal FDR	Method	$\beta^{(1)} = 0.1$				$\beta^{(1)} = 0.4$				$\beta^{(1)} = 1.6$			
		TP	FP	F1	Time (s)	TP	FP	F1	Time (s)	TP	FP	F1	Time (s)
0.05	SMA-Approx.	1.9	0.8	0.17	12	1.2	0.1	0.11	13	5.0	46.8	0.14	12
	SMA-Exact	2.0	1.0	0.17	469	1.2	0.1	0.11	507	5.0	47.0	0.14	468
	NP, $\tau = 0.348$	1.5	0.0	0.14	14	0.6	0.0	0.06	14	4.4	0.6	0.35	57
	NP, $\tau = 0.022$	2.6	0.1	0.23	19	1.8	0.1	0.16	15	4.4	0.6	0.35	62
	NP, τ estimated	2.6	0.2	0.22	31	1.6	0.2	0.15	22	4.3	0.7	0.35	72
0.1	SMA-Approx.	2.2	1.2	0.18	12	1.5	0.2	0.13	13	5.0	50.3	0.13	12
	SMA-Exact	2.3	1.4	0.19	469	1.5	0.3	0.14	507	5.0	50.6	0.13	468
	NP, $\tau = 0.348$	1.9	0.1	0.17	14	0.9	0.0	0.08	14	4.5	0.5	0.36	60
	NP, $\tau = 0.022$	3.1	0.2	0.27	23	2.2	0.2	0.19	17	4.3	0.7	0.35	62
	NP, τ estimated	3.1	0.3	0.26	33	2.3	0.2	0.20	30	4.3	0.7	0.35	70

Table S7: Results for GWAS data simulated from a LMM with $n = 400$, $p = 800,000$, $\kappa = 1$, and $\sigma^2 = 0.2$. In this table, there are 20 causal SNPs. The regression coefficients of the 20 causal SNPs are $\boldsymbol{\beta} = (\beta^{(1)}, 0.4, 0.4, 0.4, \beta^{(1)}, 0.4, 0.4, 0.4, \beta^{(1)}, 0.4, 0.4, 0.4, \beta^{(1)}, 0.4, 0.4, 0.4, \beta^{(1)}, 0.4, 0.4, 0.4)^\top$. TP indicates Average number of True Positives, FP is Average number of False Positives, and F1 is the Average F1 score. Average Performance of each method over 50 datasets for each setting.

Nominal FDR	Method	$\beta^{(1)} = 0.1$				$\beta^{(1)} = 0.4$				$\beta^{(1)} = 1.6$			
		TP	FP	F1	Time (s)	TP	FP	F1	Time (s)	TP	FP	F1	Time (s)
0.05	SMA-Approx.	0.7	0.3	0.07	13	0.5	0.1	0.05	12	5.0	44.2	0.15	12
	SMA-Exact	0.8	0.3	0.08	435	0.6	0.1	0.05	468	5.0	44.5	0.14	449
	NP, $\tau = 0.348$	0.5	0.0	0.05	14	0.3	0.0	0.02	14	4.5	0.5	0.36	59
	NP, $\tau = 0.022$	1.1	0.2	0.10	16	0.8	0.1	0.07	14	4.3	0.7	0.35	64
	NP, τ estimated	0.9	0.2	0.09	21	0.6	0.1	0.05	21	4.4	0.6	0.35	71
0.1	SMA-Approx.	1.0	0.5	0.09	13	0.8	0.3	0.07	12	5.0	47.7	0.14	12
	SMA-Exact	1.1	0.6	0.10	435	0.8	0.3	0.07	468	5.0	48	0.14	449
	NP, $\tau = 0.348$	0.7	0.1	0.06	14	0.4	0.0	0.03	14	4.4	0.6	0.36	59
	NP, $\tau = 0.022$	1.5	0.3	0.13	16	1.0	0.2	0.09	14	4.2	0.8	0.34	62
	NP, τ estimated	1.3	0.2	0.12	21	0.8	0.1	0.08	22	4.2	0.8	0.34	67

Table S8: Results for GWAS data simulated from a linear model with $n = 2,772$, $p = 800,000$, and $\sigma^2 = 0.2$. In this table, there are 20 causal SNPs. The regression coefficients of the 20 causal SNPs are $\boldsymbol{\beta} = (\beta^{(1)}, 0.4, 0.4, 0.4, \beta^{(1)}, 0.4, 0.4, 0.4, \beta^{(1)}, 0.4, 0.4, 0.4, \beta^{(1)}, 0.4, 0.4, 0.4, \beta^{(1)}, 0.4, 0.4, 0.4)^\top$. TP indicates Average number of True Positives, FP is Average number of False Positives, and F1 is the Average F1 score. Average Performance of each method over 50 datasets for each setting.

Nominal FDR	Method	$\beta^{(1)} = 0.1$				$\beta^{(1)} = 0.4$				$\beta^{(1)} = 1.6$			
		TP	FP	F1	Time (s)	TP	FP	F1	Time (s)	TP	FP	F1	Time (s)
0.05	SMA-Exact	14.1	179.2	0.13	124	18.8	257.0	0.13	125	9.0	287.9	0.06	122
	NP, $\tau = 0.348$	13.4	0.8	0.78	219	16.8	2.1	0.86	281	8.4	0.6	0.58	204
	NP, $\tau = 0.022$	14.3	1.2	0.8	235	17.0	2.2	0.87	297	10.4	1.4	0.65	243
	NP, τ estimated	14.3	1.4	0.80	244	16.7	2.9	0.84	319	10.9	1.4	0.68	256
0.1	SMA-Exact	14.2	187.3	0.13	124	18.9	269.1	0.12	125	9.2	300.3	0.06	122
	NP, $\tau = 0.348$	13.4	1.1	0.78	234	16.7	2.3	0.85	286	8.3	1.0	0.57	212
	NP, $\tau = 0.022$	14.2	1.6	0.79	246	17.1	2.2	0.87	310	11.1	1.3	0.68	251
	NP, τ estimated	14.2	2.0	0.79	258	17.3	2.5	0.87	336	11.7	1.2	0.71	275

Table S9: Results for GWAS data simulated from a LMM with $n = 2,772$, $p = 800,000$, $\kappa = 1$, and $\sigma^2 = 0.2$. In this table, there are 20 causal SNPs. The regression coefficients of the 20 causal SNPs are $\boldsymbol{\beta} = (\beta^{(1)}, 0.4, 0.4, 0.4, \beta^{(1)}, 0.4, 0.4, 0.4, \beta^{(1)}, 0.4, 0.4, 0.4, \beta^{(1)}, 0.4, 0.4, 0.4, \beta^{(1)}, 0.4, 0.4, 0.4)^\top$. TP indicates Average number of True Positives, FP is Average number of False Positives, and F1 is the Average F1 score. Average Performance of each method over 50 datasets for each setting.

Nominal FDR	Method	$\beta^{(1)} = 0.1$				$\beta^{(1)} = 0.4$				$\beta^{(1)} = 1.6$			
		TP	FP	F1	Time (s)	TP	FP	F1	Time (s)	TP	FP	F1	Time (s)
0.05	SMA-Approx.	13.6	143.3	0.15	112	17.9	193.1	0.15	100	8.9	253.9	0.06	95
	SMA-Exact	13.6	143.6	0.15	1107	17.9	193.1	0.15	1046	8.9	253.9	0.06	1710
	NP, $\tau = 0.348$	12.5	1.1	0.75	275	15.7	2.2	0.83	317	8.2	0.8	0.56	273
	NP, $\tau = 0.022$	13.1	1.6	0.75	295	16.4	2.6	0.84	329	9.8	1.5	0.62	304
	NP, τ estimated	13.2	2.3	0.74	300	15.9	3.4	0.81	345	10.3	1.7	0.64	322
0.1	SMA-Approx.	13.8	149.2	0.15	112	18.0	204.0	0.15	100	9.0	264.8	0.06	95
	SMA-Exact	13.8	149.6	0.15	1107	18.0	204.0	0.15	1046	9.0	264.8	0.06	1710
	NP, $\tau = 0.348$	12.5	1.4	0.73	274	15.8	2.4	0.83	312	8.4	1.0	0.57	284
	NP, $\tau = 0.022$	13.2	1.9	0.75	291	16.2	2.9	0.83	326	10.6	1.8	0.65	313
	NP, τ estimated	13.5	2.3	0.76	296	16.1	3.4	0.81	361	11.1	1.8	0.67	329
